# Evaluating the predictive efficacy of first trimester biochemical markers (PAPP-A, fβ-hCG) in forecasting preterm delivery incidences

**DOI:** 10.1038/s41598-024-67300-6

**Published:** 2024-07-13

**Authors:** G. Swiercz, A. Zmelonek-Znamirowska, K. Szwabowicz, J. Armanska, K. Detka, M. Mlodawska, J. Mlodawski

**Affiliations:** 1 Jan Kochanowski University, Kielce, Poland; 2 Clinic of Obstetrics and Gynecology, Provincial Combined Hospital in Kielce, Kielce, Poland

**Keywords:** Predictive markers, Prognostic markers, Ultrasonography

## Abstract

In this investigation, we explored the correlation between first-trimester biochemical markers and the incidence of preterm birth (PTB), irrespective of the cause, spontaneous preterm birth (sPTB), and preterm premature rupture of membranes (pPROM) within a cohort comprising 1164 patients. It was discovered that diminished levels of Pregnancy-Associated Plasma Protein-A (PAPP-A) between 11 and 13 + 6 weeks of gestation significantly contributed to the risk of preterm deliveries both before 35 and 37 weeks, as well as to pPROM instances. Furthermore, women experiencing sPTB before the 37th week of gestation also exhibited lower concentrations of PAPP-A. Moreover, reduced first-trimester concentrations of free beta-human chorionic gonadotropin (fb-HCG) were identified as a risk factor for deliveries preceding 37 weeks, pPROM, and sPTB before 35 weeks of gestation. Despite these correlations, the area under the curve for these biochemical markers did not surpass 0.7, indicating their limited diagnostic potential. The most significant discriminatory capability was noted for PAPP-A levels, with a threshold of < 0.71 multiples of the median (MoM) predicting PTB before 37 weeks, yielding an odds ratio of 3.11 (95% Confidence Interval [CI] 1.97–4.92). For sPTB, the greatest discriminatory potential was observed for PAPP-A < 0.688, providing an OR of 2.66 (95% CI 1.51–4.66). The cut-off points corresponded to accuracies of 76.05% and 79.1%, respectively. In regression analyses, the combined predictive models exhibited low explanatory power with R^2^ values of 9.2% for PTB and 7.7% for sPTB below 35 weeks of gestation. In conclusion, while certain biochemical markers demonstrated associations with outcomes of preterm birth, their individual and collective predictive efficacies for foreseeing such events were found to be suboptimal.

## Introduction

Preterm birth (PTB) annually impacts approximately 15 million children globally, leading to the death of one million infants each year due to complications arising from prematurity^1^. The incidence of premature births exhibits significant variability across countries, with figures ranging from 7.8% in high-income nations to 12% in low-income countries. Notably, the PTB rate within a specific population is not exclusively determined by economic factors^1^. Prematurity is associated with a wide array of potential complications, some of which are irreversible and can profoundly affect an individual's entire lifespan. Furthermore, the complications arising from prematurity impose considerable demands on healthcare systems due to the necessity for multi-specialty care. Additionally, prematurity generates psychological stress for caregivers and financial strains on nations, as it is linked to childcare requirements, social benefits, and the absence from work of parents and caregivers^2^.

Given these challenges, researchers have been actively seeking risk factors that could serve as initial indicators to categorize pregnant women into higher risk groups for PTB. To date, the literature identifies the most potent risk factors for preterm birth as a history of PTB, being under 20 years of age, low socioeconomic status, tobacco use, a Body Mass Index (BMI) lower than 19 kg/m^2^, twin pregnancy, uterine anomalies, among others^3,4^. Efforts to integrate predictive factors into comprehensive models have been made; however, these endeavors have not been entirely satisfactory due to limited reproducibility of studies and a lack of transparency^5^.

In the literature, increasing attention has been devoted to the link between the placenta and preterm birth. Literature indicates altered gene expression and production of placental proteins in cases of PTB^6^, as well as the role of placental insufficiency in their etiology^7^. Currently, in clinical settings, during the first trimester of pregnancy, we can routinely analyze the concentrations of such placental proteins as PAPP-A, PlGF (placental growth factor), and free β-hCG. However, the correlation of their concentrations with PTB as an outcome is unclear. In an attempt to address this gap, we conducted the present study.

Certain interventions have demonstrated a protective effect against PTB in patients deemed at increased risk^8^. Nevertheless, a substantial proportion of PTBs occur in women who were not previously identified as high risk^9^. Therefore, the identification of new risk factors, independent of existing ones, is imperative to enhance the data pool available for training predictive models.

An ideal scenario would involve a marker that is already being measured during pregnancy, thereby not imposing additional financial burdens on the healthcare system. Should such a marker’s predictive efficacy be established, it could independently or within a multi-factor model, classify patients into a high-risk group for PTB. Currently, all pregnant women are advised to undergo first-trimester screening, which includes a medical interview, ultrasound examination, and the evaluation of free beta-HCG (fbHCG) and pregnancy-associated plasma protein-A (PAPP-A). In this study, we aim to explore how biochemical tests, utilized in assessing the risk for non-hereditary chromosomal abnormalities during the first trimester, correlate with the risk of premature birth.

## Material and methods

### Study design and participants

All female patients who underwent a first-trimester screening test at the Department of Obstetrics and Gynecology of the Provincial Integrated Hospital in Kielce between January 2019 and December 2021 and subsequently gave birth at the clinic with complete follow-up were included in this study. Excluded from the study were patients who miscarried before the 22nd week of pregnancy or were diagnosed with a chromosomal defect. We received approval to examine the patients for research purposes from the bioethics commission at Jan Kochanowski University in Kielce (approval number—51/2023). All methods were performed in accordance with the relevant local regulations and guidelines of the ethical commission. All participants gave informed consent for ultrasonographic diagnosis and participation in the study.

### Procedure and data collection

All screenings were performed in accordance with the Fetal Medicine Foundation (FMF) guidelines^10^ and were based on medical interviews, ultrasonographic examination, and a biochemical analysis assessing the concentrations of fb-HCG and PAPP-A. Blood samples for biochemical testing were collected between the 11th and 13 + 6 weeks of gestation (gestational age adjusted based on ultrasound if it did not match the age calculated from the last menstrual period) on the same day the screening was performed. No biochemical tests were performed before the 11th week of pregnancy. All assays were conducted using the BRAHMS Kryptor analyzer. The calculation of multiples of the medians (MoM) was done using FMF 2012 software (version 3.0).

### Outcome measures

The endpoints of this study were delineated as preterm birth (PTB) occurring before 37 weeks of gestation, birth prior to 35 weeks due to its correlation with a more adverse prognosis, and premature rupture of membranes before the culmination of the 37th week of gestation (pPROM), regardless of whether this event precipitated preterm birth or necessitated the induction of labor at term. Analogously, we conducted analyses on instances of spontaneous preterm birth (sPTB) occurring before both the 37th and 35th weeks of gestation.

### Statistical analysis

For continuous variables with distributions approximating normal, the central tendency was represented using the mean. For distributions deviating from normal, the median was used to describe the central tendency. As measures of dispersion, we employed standard deviation (SD) and interquartile range (IQR) respectively. For categorical variables, percentages were used. Continuous variables across groups were compared using the Mann–Whitney U test. Differences were considered statistically significant at *p* < 0.05. For continuous predictors, the receiver operating characteristic curve (ROC curve) was plotted and the area under the curve (AUC) was calculated with a 95% confidence index (CI). Classifying cutoff points were plotted using Youden’s method. Diagnostic test parameters and odds ratios (OR) for our cohort were calculated based on these cutoffs. Logistic regression models for preterm birth < 35 and < 37 weeks of gestation were subsequently computed using the Wald forward selection method. Variables were included in the model if *p* < 0.05. Statistical analysis was carried out using Statistica 13.1 (TIBCO Software Inc.) and IBM SPSS Statistics version 26 software.

## Results

In our cohort, we included 1164 patients. The demographic characteristics are presented in Table 1.

**Table 1:** Demographic characteristics of the group.

Parameter	Value
Height [mean]	165 cm (SD = 13.33)
Weight [mean[	66 kg (SD = 7.88)
BMI [mean]	24.82 kg/m2 (SD = 4.13)
PTL (< 37 weeks of gestation)	7.21% (n = 84)
PTL (< 35 weeks of gestation)	2.57% (n = 30)
Spontaneus PTL [% in cohort]	4.63% (n = 64)
Spontaneus PTL [% in PTL patients]	76.20%
Previous preterm labour	1.55%(n = 18)
IVF pregnancy	4.3% (n = 50)
1st pregnancy	37.8% (n = 440)
Pre-pregnancy diabetes	1.12% (n = 13)
Pre-pregnancy hypertension	0.6% (n = 7)
High risk for trisomy 21 (> 1:300)	7.98% (n = 93)
Intermediate risk for trisomy 21 (1:301–1:1000)	14% (n = 163)
High risk for trisomy 18 (> 1:300)	0.68% (n = 8)
Intermediate risk for trisomy 18 (1:301–1:1000)	1.97% (n = 1164)
High risk for trisomy 13 (> 1:300)	0.51% (n = 6)
Intermediate risk for trisomy 13 (1:301–1:1000)	0.77% (n = 9)
PAPP-A < 0,4 MoM	3.78% (n = 44)

*PTL*—preterm labour, IVF—in vitro fertilization.

The incidence of preterm births occurring before 35 weeks of gestation was recorded at 2.57% (encompassing 30 cases), whereas the incidence of preterm births occurring before 37 weeks of gestation stood at 7.21% (totaling 84 cases). Within this cohort, 76.2% of the preterm births were categorized as spontaneous, with the remaining instances being attributed to iatrogenic causes. The occurrence rate of patients who experienced pPROM was documented at 4.46% (accounting for 52 cases). We embarked on a comparison of the median values of laboratory parameters during the first trimester of pregnancy among the groups of patients who delivered before 35 weeks, before 37 weeks, and those who encountered pPROM against the remainder of the cohort. Subsequently, a similar analysis was performed, this time excluding patients who underwent preterm delivery due to iatrogenic reasons, thereby focusing solely on cases of sPTB. The comparisons encompassed both the median absolute values and the coefficients normalized for the specific population, adhering to the methodology advocated by the Fetal Medicine Foundation (FMF). The outcomes of these comparisons are elucidated in Table 2.

**Table 2:** Comparison of medians of biochemical parameters in different groups.

Parameter	Median	IQR	Median	IQR	*p*
	< 35 weeks		> = 35 weeks		
fbHCG	32.1050	12.84000	39.7450	32.10000	0.174182
fbHCG MoM	1.0230	0.45100	1.1115	0.86700	0.692786
PAPP-A	3.2600	2.53000	3.8750	3.21500	0.095418
PAPP-A MoM	0.8285	0.47900	1.0240	0.68400	0.018235
	< 37 weeks		> = 37 weeks		
fbHCG	32.4250	19.33000	40.0450	32.16500	0.006876
bhcg MoM	0.9470	0.57000	1.1200	0.87800	0.022779
PAPP-A	2.7100	2.36000	3.9600	3.19000	0.000023
PAPP-A MoM	0.7780	0.56600	1.0370	0.67600	0.000009
	pPROM		no-pPROM		
fbHCG	31.7600	16.98500	39.9600	32.12000	0.001997
bHCG MoM	0.9070	0.55200	1.1170	0.88000	0.016850
PAPP-A	3.1700	2.58000	3.9200	3.20000	0.014168
PAPP-A MoM	0.8225	0.60450	1.0280	0.68500	0.002487
*Spontaneus preterm birth*					
	< 35 weeks		> = 35 weeks		
fbHCG	30.28000	11.00000	39.93500	32.14500	0.032157
fbHCG MoM	1.02300	0.43300	1.11400	0.86800	0.353608
PAPP-A	3.51000	2.27000	3.92000	3.20000	0.431336
PAPP-A MoM	0.91150	0.66500	1.02800	0.67700	0.109673
	< 37 weeks		> = 37 weeks		
fbHCG	30.39500	16.19000	40.08500	32.06500	0.004309
fbHCG MoM	0.97450	0.56700	1.11800	0.88000	0.037868
PAPP-A	3.05500	2.83000	3.95000	3.19000	0.011908
PAPP-A MoM	0.85300	0.65300	1.03700	0.67800	0.003966

*IQR*—interquartile range.

For women who delivered prematurely before the 35th week of gestation, there was a statistically significant reduction in the median value of normalized PAPP-A (MoMs). In the case of patients with PTB before the 37th week of gestation and for those who experienced pPROM, a statistically significant disparity was observed in all biochemical markers. fbHCG concentrations and PAPP-A levels, in both absolute and normalized terms, were lower in instances involving the aforementioned complications. Subsequently, an analysis was conducted exclusively for sPTB patients. Considering the outcome of birth before 35 weeks of gestation, a diminished concentration of fb-HCG in absolute values was noted among women with spontaneous preterm birth. Similarly, for sPTB before the 37th week of gestation, a reduction in all parameters measured in the first trimester of pregnancy was observed.

Given the differences identified, Receiver Operating Characteristic (ROC) curves were plotted for all parameters that exhibited significant variations between the groups and for individual outcomes. Applying Youden’s principle, cutoff points were established to optimize the sensitivity and specificity ratio. The findings of this analytical process are detailed in Table 3.

**Table 3:** Parameters describing the plotted ROC curves, along with statistical significance and the cutoff point determined by Youden’s method.

Predictor	AUC	AUC low CI 95%	AUC high CI 95%	*p*	Cut-off point
< 35					
PAPP-A MoM	0.626	0.534	0.718	0.0071	1.18
< 37					
fbHCG	0.595	0.553	0.658	0.0057	41.16
bHCG MoM	0.575	0.511	0.639	0.0225	1.32
PAPP-A	0.638	0.579	0.696	0.0000	3.39
PAPP-A MoM	0.0643	0.581	0.706	0.0000	0.71
pPROM					
fbHCG	0.627	0.557	0.697	0.0004	40.06
bHCG MoM	0.598	0.526	0.67	0.0077	1.3
PAPP-A	0.601	0.526	0.675	0.0085	3.96
PAPP-A MoM	0.624	0.546	0.702	0.0019	0.67
Spontaneus preterm labour					
< 37 weeks of gestation					
fbHCG	0.615	0.542	0.688	0.0021	26.65
bHCG MoM	0.584	0.508	0.659	0.0296	1.302
PAPP-A	0.601	0.526	0.676	0.0080	3.38
PAPP-A MoM	0.616	0.534	0.698	0.0056	0.688
< 35 weeks of gestation					
fbHCG	0.401	0.273	0.528	0.1275	40.93

*CI*—confluence index, *AUC*—area under curve.

The area under the curve (AUC) for any investigated parameter did not exceed 0.7, implying that none of the parameters were sufficiently reliable for use in the differential diagnostic process between conditions. The highest AUC values were observed for predictors of the occurrence of pPROM, yet all values remained below the 0.7 threshold. In the case of sPTB occurring before 35 weeks of gestation as an outcome, the AUC did not significantly differ from 0.5 (*p* = 0.13), suggesting no better predictive value than chance.

The Youden index points determined from the ROC curves were utilized to establish the cutoff parameter for the diagnostic test within our cohort. Table 4 elucidates the diagnostic efficacy of the tests for the computed cutoff points, along with the odds ratios (OR) for the occurrence of the complication when the cutoff criterion is met. The analysis excluded spontaneous preterm births < 35 weeks of gestation due to an AUC < 0.5, indicating inadequate predictive capability.

**Table 4:** Diagnostic performance of potential dichotomous tests based on meeting the cut-off criteria determined on ROC curves.

Parameter	OR	95% CI	Sensitivity (95%CI)	Specificity	PPV	NPV	Accuracy
PTB < 35 weeks of gestation							
PAPP-A MoM < 1.18	4.27	1.48–12.34	86.67%	39.70%	3.65%	99.12%	40.90°%
PTB < 37 weeks of gestation							
PAPP-A < 3.39	2.8	1.74–4.49	65.85%	59.22%	10.90%	95.82%	59.69%
PAPP-A MoM < 0.71	3.11	1.97–4.92	46.34%	78.30%	13.92%	95.07%	76.05%
Free bHCG < 41.16	2.57	1.55–4.26	73.17%	50.00%	9.98%	96.10%	40.31%
Free bHCG MoM < 1.31	2.12	1.24–3.64	78.05%	37.45%	8.63%	95.75%	40.31%
pPROM							
bHCG < 40.06	3.69	1.87–7.26	78.85%	49.77%	6.83%	98.05%	51.07%
bHCG MoM < 1.3	2.58	1.28–5.20	80.77%	38.10%	5.74%	97.70%	40.01%
PAPP-A < 3.96	2.409	1.30–4.44	71.15%	49.41%	6.16%	97.35%	50.38%
PAPP-A MoM < 0.67	2.76	1.55–4.9	40.38%	80.33%	8.75%	96.65%	78.55%
Spontaneus preterm labour							
PTB < 37 weeks of gestation							
Free bHCG < 26.65	8.0364	4.36–14.81	27.80%	75.60%	5.40%	95.40%	24.40%
bHCG MoM < 1.302	2.1611	1.12–4.15	79.60%	38.20%	6.10%	97.40%	40.20%
PAPP-A < 3.38	1.968	1.13–3.42	59.30%	59.20%	6.80%	96.70%	59.20%
PAPP-A MoM < 0.688	2.6567	1.51–4.66	38.90%	81.10%	9.40%	96.40%	79.10%

*OR*—odds ratio, *CI*—confidence index, *PPV*—positive predictive value, *NPV*—negative predictive value.

The assessment revealed that the test with the highest diagnostic accuracy was identified for the concentration of PAPP-A < 0.71 MoM. At this cutoff point, the OR for a preterm birth occurring before 37 weeks of gestation was 3.11 (95% CI 1.97–4.92), denoting a high negative predictive value and favorable specificity. The greatest diagnostic accuracy for the prediction of pPROM was achieved with a PAPP-A concentration of < 0.67 MoM, with an accuracy rate of 78.55%, characterized by substantial specificity and a high negative predictive value (NPV). For preterm births occurring before 35 weeks, the determined PAPP-A MoM cutoff value did not demonstrate satisfactory accuracy. In cases of spontaneous preterm birth before 37 weeks, the highest accuracy was observed with PAPP-A MoM, where a cutoff point of < 0.668 resulted in an accuracy of 79.10%.

Subsequent to the initial analyses, we executed a logistic regression analysis employing the Wald stepwise selection method, detailed in Table 5. This analysis incorporated the biochemical parameters identified in the first trimester, alongside potential confounding variables such as height, weight, and BMI—both in quantitative and qualitative assessments—and data from interviews, including PE, pre-pregnancy diabetes, pre-pregnancy hypertension, pregnancies after in vitro fertilization (IVF), smoking status, and a history of preterm birth. Two explanatory variables were included in the predictive model for births occurring before 37 weeks of gestation—BMI and a history of preterm birth. For PTB before 35 weeks of gestation, the model incorporated PAPP-A MoM and BMI. A similar analysis was conducted for sPTB. In the model predicting sPTB before 37 weeks, the only significant predictor was a history of preterm birth, while for the prediction of sPTB before 35 weeks of gestation, the model included both absolute and normalized fb-HCG levels.

**Table 5:** Logistic regression analyses for specific outcomes.

Parameter	B	Standard error	Wald	df	*p*	Exp(B)
PTB < 37 weeks of gestation						
BMI (kg/m^2^)	0.009534974	0.004472903	4.544226151	1	0.0330299	1.009580576
History of preterm birth	− 1.928875978	0.627047451	9.46254146	1	0.0020971	0.14531144
Constant	− 2.74283187	0.453585491	36.5662199	1	1.476E-09	0.064387751
R^2^ = 7.3%						
PTB < 35 weeks of gestation						
PAPP-A MoM (IU/L)	− 1.82891807	0.94748585	3.726001589	1	0.0535715	0.160587218
BMI (kg/m^2^)	0.015952975	0.005715892	7.789602036	1	0.0052548	1.016080903
Constant	0.329589273	1.621064984	0.041337603	1	0.8388877	1.390396937
R^2^ = 9.2%						
Spontaneus PTB < 37 weeks of gestation						
History of preterm birth	1.57170964	0.804428386	3.817419251	1	0.0487223	4.814872858
R^2^ = 4.2%						
Spontaneus PTB < 35 weeks of gestation						
fbHCG	− 0.072580085	0.02682553	7.320459131	1	0.0068174	0.929991266
fbHCG MoM	1.593878529	0.62691655	6.463845549	1	0.0110091	4.922805194
Constant	− 2.542950459	1.800319911	1.995154029	1	0.157803	0.078634051
R^2^ = 7.7%						

*B*—regression coefficient.

However, the performance of all models was found to be suboptimal, with the coefficient of determination (R^2^) for all models ranging from 4.2 to 9.2%. Biochemical parameters were not included in the predictive model for sPTB and PTB before 37 weeks of gestation when a history of preterm birth was present as a cofactor. This outcome suggests that, although certain individual and combined factors were statistically significant, their overall predictive value within the models remained limited, highlighting the complexity of predicting preterm birth outcomes and the potential need for integrating additional or alternative predictors to enhance model efficacy.

## Discussion

Biochemical studies are an essential aspect of evaluating the risk of non-hereditary chromosomal anomalies during the first trimester of pregnancy, which is universally recommended for all expectant mothers^10,11^. One of the key biomarkers in this screening is PAPP-A, a protease that plays a critical role in liberating Insulin-like Growth Factors (IGFs) from their binding proteins, thus enabling these growth factors to bind to the IGF receptor 1^12^. Beyond its utility in assessing the risk for chromosomal abnormalities, PAPP-A is also instrumental in evaluating the potential for PE and fetal growth restriction (FGR). Furthermore, research has indicated a correlation between PAPP-A levels and the risk of developing gestational diabetes mellitus (GDM) and the placenta accreta spectrum (PAS), highlighting its significance in prenatal diagnostics^12^.

Outside the realm of pregnancy, PAPP-A has been investigated for its association with cardiovascular risk, underscoring its broad relevance to human health^13^. The literature describes its involvement in a variety of physiological and pathological processes, including metabolism, cancer, and neurodegenerative diseases^14^. As a regulator of IGF-1 activity, PAPP-A has been implicated in the pathogenesis of conditions like gigantism and acromegaly^15^. Additionally, emerging evidence suggests a link between PAPP-A levels and the incidence of preterm birth, proposing its utility as a predictive biomarker for such outcomes^16^.

The fbHCG, an alternatively glycosylated monomeric variant of hCG^17^, is another critical biomarker. Its utility extends beyond the diagnosis of fetal chromosomal diseases, as variations in fbHCG levels have been associated with preeclampsia^18^ and preterm birth^19^, further affirming the value of these biomarkers in the comprehensive management and prognosis of pregnancy-related conditions.

Our investigation suggests that low levels of biochemical markers measured in the first trimester may serve as a risk factor for PTB, regardless of its cause, as well as specifically in cases of sPTB. The link with iatrogenic preterm birth appears intuitive, given the association of low PAPP-A levels with PE and FGR^20^. Nonetheless, research also establishes a connection with sPTB, which presently constitutes the majority of PTB instances^21^. The literature provides evidence supporting such correlations. Specifically, a PAPP-A concentration below the 10th percentile in the first trimester is associated with an increased risk of PTB before 35 weeks of gestation (adjusted OR [aOR] = 2, 95% CI 1–2.8) and before 32 weeks of gestation (aOR = 2.7, 95% CI 1.1–6.4, *p* = 0.02), even after adjusting for other risk factors, including previous PTB. However, this study did not identify it as a sufficiently reliable predictive factor (AUC = 0.63, 95% CI = 0.53–0.72)^20^.

In the research conducted by Giorgione et al.^23^, where screening for PE was based on mean arterial pressure (MAP), uterine artery pulsatility index (UtA–PI), and PAPP-A, along with the prophylactic administration of acetylsalicylic acid for patients with a PE risk greater than 1:50 (as calculated according to FMF guidelines), it was found that patients experiencing PTB (consisting of 35.2% sPTB and the remainder iatrogenic PTB) had significantly lower PAPP-A MoM levels (0.89 vs 0.92, *p* < 0.05) than those who delivered after 37 weeks of gestation. A high risk of PE (> 1:50) also increased the risk of sPTB, after the exclusion of patients with iatrogenic preterm birth (OR = 2, 95% CI = 1.46–2.86)^23^.

Furthermore, the study by Chiu et al. also demonstrated that lower PAPP-A concentrations are associated with an increased risk of sPTB before 34 and before 37 weeks of gestation, as well as pPROM^16^. Such findings may indicate a common pathological pathway leading to both sPTB and iatrogenic PTB (iPTB), suggesting that both pathways could be linked to placental insufficiency^16^.

In our patient cohort, we obtained similar results, with PAPP-A MoMs values of 0.82 versus 1.02 acting as a discriminating factor for PTB before 35 weeks of gestation, and 0.77 versus 1.03 for birth before 37 weeks of gestation. This data confirms that a low concentration of PAPP-A is a significant risk factor for PTB, irrespective of the cause. A similar distinction was observed in our study when considering women with sPTB before 37 weeks of gestation. However, the discriminatory capability of this biomarker is not sufficient for it to be utilized as an independent predictive factor or within complex models, as the findings suggest an association rather than a causative link.

In the research conducted by Ravenswaaij et al.^24^, an attempt was made to construct logistic regression models for predicting adverse pregnancy outcomes utilizing variables such as PAPP-A concentration, fb-HCG level, maternal weight, and smoking. The resulting model for predicting PTB had a coefficient of determination (R^2^) of merely 1%, with an AUC of 56%, and only 6% of patients were correctly classified by the model^24^. Compared to this, our model achieved a higher R^2^ coefficient but still only reached a maximum of 9.2% in predicting preterm birth below 35 weeks of gestation, even in a combined model that also incorporated other risk factors, such as BMI. These outcomes highlight the challenges in accurately predicting PTB and the limitations inherent in current predictive models, pointing to the essential need for ongoing research and the development of more effective predictive methodologies.

In another multifactorial model cited in the literature, PAPP-A MoM values were leveraged for the prediction of pPROM. The variables incorporated into this model included fertility issues, PAPP-A MoM levels, history of cervical conization, previous preterm birth before 37 weeks of gestation, and a cervical canal length of less than 25 mm measured in the first trimester of pregnancy. The area under the curve for this model was reported at 0.72, indicating moderate predictive accuracy. Notably, this model was able to detect 30% of patients with pPROM while maintaining a false-positive detection rate of 10%^25^.

Furthermore, an AUC value of 0.78 (95% Confidence Interval [CI] 0.69–0.88) was achieved by a predictive model that delineated preterm birth (PTB) before 34 weeks of gestation. This model integrated maternal characteristics, ADAM12 protein concentration, PAPP-A levels, and uterine artery pulsatility index (UtA PI). Despite its higher AUC, this model exhibited relatively low sensitivity, with 42% for a 10% false-positive ratio (FPR) and 65% for a 20% FPR^26^.

These findings underscore that incorporating additional risk factors alongside PAPP-A measurements can enhance the predictive capabilities of models designed to foresee PTB and pPROM. Nonetheless, despite these improvements, the models still demonstrate insufficient discriminatory power to accurately identify patients who will subsequently undergo PTB, highlighting the complex nature of PTB prediction and the need for further refinement of these predictive tools.

The literature presents limited data on the predictive abilities of free beta-human chorionic gonadotropin (fb-HCG). In the research conducted by Dane et al.^29^, fb-HCG concentration was not found to have significant predictive capabilities towards PTB before 34 weeks of gestation in normotensive women (AUC = 0.62, 95% Confidence Interval [CI] = 0.58–0.65), rendering the model statistically insignificant. This finding aligns with the results of our study. For PAPP-A within the same investigation, an AUC of 0.74 (95% CI 0.71–0.77, *p* = 0.0002) was demonstrated, indicating a higher level of predictive accuracy^27^.

Additionally, it was shown that fbHCG concentrations below 0.5 MoMs, excluding cases with fetal chromosomal or structural anomalies and underlying diseases, significantly increased the risk for FGR (Relative Risk [RR] = 1.66, 95% CI = 1.22–2.53), PTB (RR = 1.43, 95% CI = 1.10–1.87), low birth weight (LBW) (RR = 1.83, 95% CI = 1.46 – 2.29), and a low Apgar score (RR = 2.89, 95% CI = 1.73–4.85). In contrast, higher fbHCG levels were associated with a decreased risk of PTB (RR = 0.73, 95% CI = 0.56–0.95) and GDM (RR = 0.62, 95% CI = 0.45–0.84)^28^. Efforts to enhance the predictive capability by integrating fb-HCG with other markers were explored; for instance, Celik et al.^27^ examined the ratio of alpha-fetoprotein (AFP) concentration to fb-HCG, finding that a ratio > 0.6 significantly increased the risk for spontaneous PTB (sPTB) ≤ 32 weeks by 3.5 times.

In our research, fb-HCG levels were significantly lower in pregnancies that concluded before 37 weeks of gestation and in cases of pPROM. For sPTB, we observed decreased fb-HCG concentrations in instances of birth before 35 weeks, as well as sPTB before 37 weeks of gestation. The cutoff point determined using the Youden method in our study showed a very high value, covering more than two quartiles of the concentration distribution, which made it impractical for clinical use.

A significant limitation of our investigation is its retrospective design. Nevertheless, considering the limited effectiveness of the predictive models identified, there seems to be little rationale in validating these models through a prospective study. The cut-off points determined by our research should, therefore, be interpreted merely as risk indicators for this specific adverse obstetric outcome. Another constraint is the absence of adjustments for numerous independent risk factors for preterm birth (PTB) that are well-documented in the literature, such as low socioeconomic status, cervical insufficiency, history of cervical surgery, and the use of illicit drugs and alcohol.

Conversely, a notable strength of our study is the considerable size of the patient cohort, which is substantial by Polish standards, enhancing the relevance and applicability of our findings within this demographic context. Additionally, the consistency of laboratory and clinical assessments across the cohort—attributable to all women delivering in the same medical center—reinforces the reliability and repeatability of our results, providing a solid foundation for future research endeavors aimed at refining PTB predictive models.

## Conclusion

Our findings reveal that low levels of PAPP-A and fb-HCG in the first trimester are associated with an increased risk of preterm birth before 35 and 37 weeks, pPROM, and sPTB before 37 weeks. However, the predictive power of these biochemical markers, both individually and in combined models, is insufficient for reliable preterm birth prediction. This highlights the need for further research to enhance predictive accuracy.

## Data Availability

The dataset used for this study was uploaded to a public repository and is available at this URL: 10.17605/OSF.IO/XZ8RQ.
